# Effect of an Introduced Phytoene Synthase Gene Expression on Carotenoid Biosynthesis in the Marine Diatom *Phaeodactylum tricornutum*

**DOI:** 10.3390/md13085334

**Published:** 2015-08-20

**Authors:** Takashi Kadono, Nozomu Kira, Kengo Suzuki, Osamu Iwata, Takeshi Ohama, Shigeru Okada, Tomohiro Nishimura, Mai Akakabe, Masashi Tsuda, Masao Adachi

**Affiliations:** 1 Laboratory of Aquatic Environmental Science, Faculty of Agriculture, Kochi University, Otsu-200, Monobe, Nankoku, Kochi 783-8502, Japan; E-Mails: kadono-takashi@kochi-u.ac.jp (T.K.); aquariumuirauqa@gmail.com (T.N.); 2 The United Graduate School of Agricultural Sciences, Ehime University, 3-5-7 Tarumi, Matsuyama, Ehime 790-8566, Japan; E-Mail: s10dre05@s.kochi-u.ac.jp; 3 Euglena Co., Ltd., 4th Floor, Yokohama Leading Venture Plaza, 75-1 Ono-cho, Tsurumi-ku, Yokohama, Kanagawa 230-0046, Japan; E-Mails: suzuki@euglena.jp (K.S.); iwata@euglena.jp (O.I.); 4 School of Environmental Science and Engineering, Kochi University of Technology, Tosayamada, Kami, Kochi 782-8502, Japan; E-Mail: ohama.takeshi@kochi-tech.ac.jp; 5 Department of Aquatic Biosciences, The University of Tokyo, 1-1-1, Yayoi, Bunkyo-ku, Tokyo 113-8657, Japan; E-Mail: aokada@mail.ecc.u-tokyo.ac.jp; 6 Synthetic Organic Chemistry Laboratory, RIKEN, 2-1 Hirosawa, Wako, Saitama 351-0198, Japan; E-Mail: mai.akakabe@riken.jp; 7 Science Research Center, Kochi University, Oko-cho Kohasu, Nankoku, Kochi 783-8506, Japan; E-Mail: mtsuda@kochi-u.ac.jp; 8 Center for Advanced Marine Core Research, Kochi University, Otsu-200, Monobe, Nankoku, Kochi 783-8502, Japan

**Keywords:** microalgae, marine diatom, transformation, PSY, carotenoid

## Abstract

Carotenoids exert beneficial effects on human health through their excellent antioxidant activity. To increase carotenoid productivity in the marine Pennales *Phaeodactylum* *tricornutum*, we genetically engineered the phytoene synthase gene (*psy*) to improve expression because RNA-sequencing analysis has suggested that the expression level of *psy* is lower than other enzyme-encoding genes that are involved in the carotenoid biosynthetic pathway. We isolated *psy* from *P. tricornutum*, and this gene was fused with the enhanced green fluorescent protein gene to detect *psy* expression. After transformation using the microparticle bombardment technique, we obtained several *P. tricornutum* transformants and confirmed *psy* expression in their plastids. We investigated the amounts of PSY mRNA and carotenoids, such as fucoxanthin and β-carotene, at different growth phases. The introduction of *psy* increased the fucoxanthin content of a transformants by approximately 1.45-fold relative to the levels in the wild-type diatom. However, some transformants failed to show a significant increase in the carotenoid content relative to that of the wild-type diatom. We also found that the amount of PSY mRNA at log phase might contribute to the increase in carotenoids in the transformants at stationary phase.

## 1. Introduction

Diatoms are unicellular photosynthetic eukaryotes with an estimated 200,000 species in ocean, fresh water and in/on soil [1]. Diatoms are of broad interest for basic studies of the ecosystem, evolution and metabolism due to their enormous contribution to primary production on Earth [2], complex evolutionary history as secondary endosymbionts [3] and their unique ability to produce silica-based cell walls [4]. In addition, diatoms represent a potential source for commercial and industrial applications, because they produce various beneficial chemicals for human activities, such as polyunsaturated fatty acids, vitamins, antioxidants, enzymes, polysaccharides and carotenoids [5].

The molecular biology technology of diatoms includes progress in genomic analysis, transcriptome analysis and genetic transformation systems. The databases of genome sequences and expressed sequence tags (ESTs) of the model diatoms Pennales *Phaeodactylum tricornutum* and Centrics *Thalassiosira pseudonana* are open to the public and result in improvements in the understanding of metabolism in diatoms [6,7,8]. In addition to model diatoms, the genome sequences of diatoms, such as *Pseudo-nitzschia multiseries* and *Fragilariopsis cylindrus* [9], have become available on the web site of the U.S. Department of Energy Joint Genome Institute (http://jgi.doe.gov/). Genetic transformation techniques have been established for several species of diatoms, such as Pennales *P. tricornutum* [10,11,12,13], *Cylindrotheca fusiformis* [14,15], *Navicula saprophila* [16], *Pseudo-nitzschia arenysensis* [17] and *Pseudo-nitzschia multistriata* [17] and Centrics *Cyclotella cryptica*, *T. pseudonana* [18], *T. weissflogii* [19], *Chaetoceros* sp. [20] *Chaetoceros gracilis* [21] and *Fistulifera* sp. [22]. Especially, *P. tricornutum* has the advantage in the field of metabolic engineering due to the powerful molecular techniques that are available for the suppression/knockdown of target genes in *P. tricornutum*, such as RNA silencing [23,24,25], and for genome editing using transcription activator-like effector nucleases (TALENs) [26,27] These molecular tools have been used in some studies to create genetically-transformed diatoms as potential sources of biofuels [28,29,30] and specialty chemicals [31,32].

Carotenoids are synthesized via the methylerythritol phosphate (MEP) pathway in all photosynthetic organisms, including diatoms, and in some non-photosynthetic organisms, such as bacteria and fungi [33]. Carotenoids make up the pigment-protein complexes that associate with chlorophyll for the formation of functional photosynthesis apparatus [34]. The major function of carotenoids is light harvesting and the transfer of the excitation energy via chlorophylls to the photosynthetic systems [35]. In addition to their participation in light-harvesting functions, carotenoids act as a key facilitator of photoprotectants by quenching the triplet excited state of chlorophyll and dissipation of the energy as heat [35]. This transfer of excitation energy results in the quenching of singlet oxygen (^1^O_2_) [36]. This ability to quench ^1^O_2_ provides the beneficial effects on human heath of avoiding oxidative conditions, thereby preventing diseases, such as cancer and cardiovascular disease [37].

**Figure 1:**
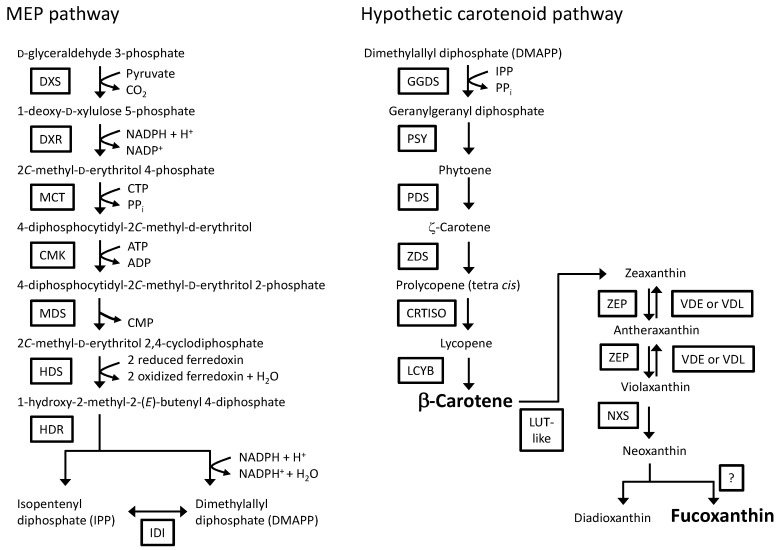
Schematic representation of the MEP pathway and the carotenoid biosynthetic pathway in *P. tricornutum*. Modified from Bertrand [38], Coesel *et al.* [39], Dambek *et al.* [40], Hemmerlin [41], Lohr *et al.* [42], Mikami and Hosokawa [43] and Vranová *et al.* [44]. DXS: 1-deoxy-d-xylulose 5-phosphate synthase; DXR: 1-deoxy-d-xylulose 5-phosphate reductoisomerase; MCT: 2*C*-methyl-d-erythritol 4-phosphate cytidyltransferase; CMK: 4-diphosphocytidyl-2*C*-methyl-d-erythritol kinase; MDS: 2*C*-methyl-d-erythritol 2,4-cyclodiphosphate synthase; HDS: 1-hydroxy-2-methyl-2-(*E*)-butenyl 4-diphosphate synthase; HDR: 1-hydroxy-2-methyl-2-(*E*)-butenyl 4-diphosphate reductase; IDI: isopentenyl diphosphate:dimethylallyl diphosphate isomerase; GGDS: geranylgeranyl diphosphate synthase; PSY: phytoene synthase; PDS: phytoene desaturase; ZDS: ζ-carotene desaturase; CRTISO: carotenoid isomerase; LCYB: lycopene β-cyclase; LUT-like: lutein deficient-like; ZEP: zeaxanthin epoxidase; VDE: violaxanthin de-epoxidase; VDL: violaxanthin de-epoxidase-like; NXS: neoxanthin synthase.

Diatoms produce a number of carotenoids, including, diatoxanthin, diadinoxanthin and fucoxanthin [45]. The carotenoids in *P. tricornutum* were investigated in detail, and the carotenoid biosynthetic pathway using dimethylallyl diphosphate as a final product synthesized in the MEP pathway has been proposed [40]. The predicted carotenoid biosynthetic pathway in *P. tricornutum* is summarized in Figure 1. In addition, *P. tricornutum* has commercial potential as a natural source of fucoxanthin, which is used in human and animal food, health and cosmetic products [46]. Several works that have focused on the overproduction of carotenoids in plants and green algae have suggested increasing the carotenoid production in *P. tricornutum*. In plants, the overexpression of bacterial or plant phytoene synthase (PSY), which is considered a rate-limiting enzyme in the carotenoid biosynthetic pathway [47], resulted in increased carotenoid production [48,49,50,51]. An increase in carotenoid production due to the expression of exogenous PSY was also observed in the unicellular alga *Chlamydomonas reinhardtii* [52,53]. The overexpression of the PSY transcript in *P. tricornutum* resulted in an increase in the amount of carotenoids and PSY mRNA [54]. Another article reported that the suppression of PSY mRNA levels with microRNA [55] reduced the carotenoid levels. These results suggest a correlation between the amount of PSY mRNA and the amount of carotenoids. In addition to altering carotenoid levels with genetic engineering of *psy*, carotenoid levels and composition can be influenced by the growth stage of *P. tricornutum* [56,57]. However, the effect of growth stage on the production of carotenoids in *psy* transformants was unclear in the studies discussed above [54,55]. Thus, in this study, a transformation system was used to engineer *P. tricornutum* to increase carotenoid accumulation by overexpressing key players in the carotenoid biosynthetic pathway. A PSY gene from *P. tricornutum* was fused to enhanced green fluorescent protein (eGFP) gene (*egfp*). The gene fusion was introduced into *P. tricornutum*, and the levels of mRNA expression and carotenoid contents in the transformants were analyzed in different growth stages.

## 2. Results

### 2.1. Phylogenetic Analyses of P. tricornutum Strain NRIA-0065

In this study, we selected the domestic strain *P. tricornutum* NRIA-0065, which is found in Aichi prefecture, Japan, for the transformation, because *P. tricornutum* adapts to the domestic ecological system and environment and was expected to have advantages for commercial use in Japan. This strain has been identified based only on the morphological features, but it has not been analyzed phylogenetically. To determine the phylogenetic position of the *P. tricornutum* strain NRIA-0065, the 18S ribosomal RNA gene (rDNA) and internal transcribed spacer regions (ITS1 and ITS2) containing the 5.8S rDNA (ITS1-5.8S rDNA-ITS2) were amplified by polymerase chain reaction (PCR) using the cells as templates with specific primer sets (Table S1). Amplicons were sequenced and the phylogenetic position was analyzed with reference sequences from GenBank. The phylogenetic analysis based on the 18S rDNA sequences showed that the strain NRIA-0065 is positioned in a monophyletic group of *P. tricornutum* (Figure S1A). The ITS1-5.8S-ITS2 tree also showed that the strain NRIA-0065 belonged to the *P. tricornutum* clade (Figure S1B).

### 2.2. RNA-Seq Analysis of P. tricornutum and Sequence Analysis of P. tricornutum PSY

**Figure 2:**
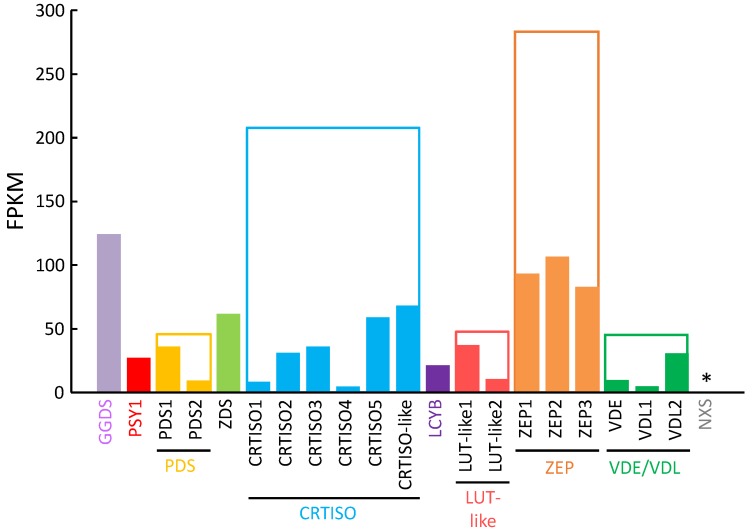
RNA-seq analysis of putative genes in the carotenoid biosynthetic pathway. FPKM: fragments per kilobase of exon per million mapped sequence reads; solid bar: FPKM of each gene; open bar: sum of FPKM of genes that are considered to belong to the same gene family; * the gene shows homology to the plant NXS gene, which was not available in our database of *P. tricornutum* strain NRIA-0065.

To select a target gene for transformation of *P. tricornutum* for carotenoid production, we compared gene expression levels of the predicted enzymes in the carotenoid biosynthetic pathway by screening the draft sequence of the *P. tricornutum* strain NRIA-0065 genome in our database and performing RNA-sequencing analysis. The expression level of the PSY gene was relatively low among the measured transcripts (Figure 2). In addition to the expression level of the PSY gene, PSY is generally thought to be the most important regulatory enzyme in the carotenoid biosynthetic pathway in plants [47]. Thus, we selected the PSY gene as a target.

**Figure 3:**
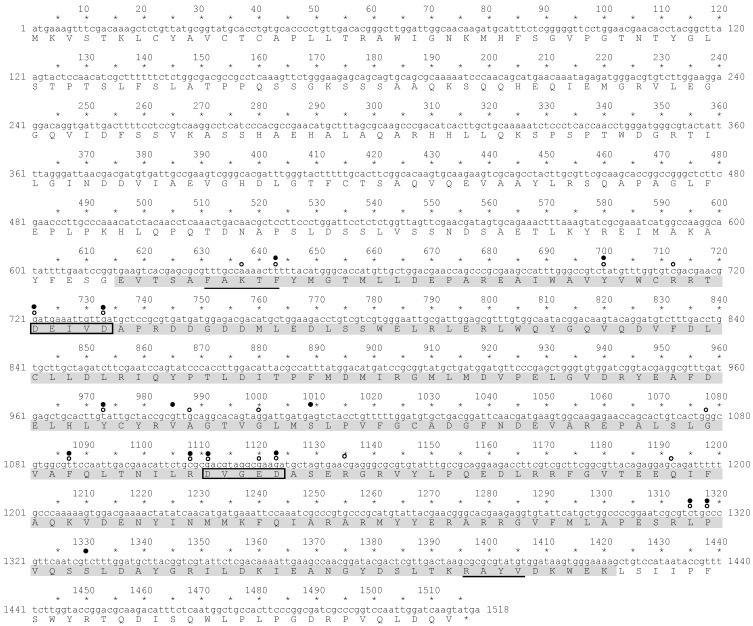
Sequence and structure of the PSY gene of *P. tricornutum* strain NRIA-0065. The amino acid sequence of PSY was analyzed by an NCBI-provided Conserved Domain Search. Highlighted in grey: homology to *trans*-isoprenyl diphosphate synthase; framed amino acids: aspartate rich regions and substrate-Mg^2+^-binding sites (DXXXD); open circle: substrate binding pocket; filled circle: catalytic residues; line: active site lid residues.

The cDNA fragment containing the potential *P. tricornutum* PSY gene was obtained by reverse transcript-PCR (RT-PCR) and then sequenced (Figure 3). *In silico* analysis of the deduced amino acid sequence (505 amino acids) from this cDNA showed there was a region that exhibited homology to a *trans*-isoprenyl diphosphate synthase domain, which is included in squalene synthase and phytoene synthase [58,59]. The *trans*-isoprenyl diphosphate synthase domain of PSY catalyzes the head to head (1′-1) condensation of two molecules of geranylgeranyl diphosphate to produce phytoene [60,61]. Four characteristic sites were identified by *in silico* analysis in the predicted domain of the potential *P. tricornutum* PSY, which included a substrate-Mg^2+^-binding site (aspartate rich region), a substrate binding pocket, catalytic residues and active site lid residues (Figure 3).

We also aligned the deduced amino acid sequence from the cDNA sequence with PSY sequences of various organisms (Figure S2). The alignment indicated that the *trans*-isoprenyl diphosphate synthase domain of *P. tricornutum* PSY had a high homology to those of the various organisms. In the plant cases, PSY proteins contained predicted chloroplast transient peptides at the *N*-terminal region that locate the proteins in the chloroplast [62]. However, in the PSY sequence of *P. tricornutum*, the *P. tricornutum* plastid-targeting cleavage site [63] was not found at the *N*-terminal side of the *trans*-isoprenyl diphosphate synthase domain (Figure 3).

### 2.3. Screening of Transformants 

**Figure 4:**
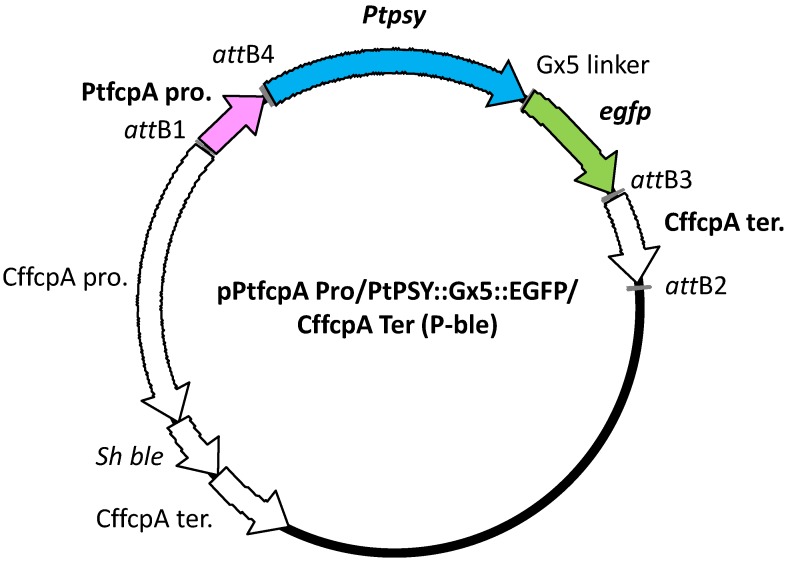
Structure of the transformation vector. Abbreviations used in this map: PtfcpA pro.: the promoter region of FCP gene A derived from *P. tricornutum* UTEX 646; *Ptpsy*: *P. tricornutum* PSY gene isolated from *P. tricornutum* NRIA-0065; *egfp*: eGFP gene; CffcpA ter.: the terminator region of FCP gene derived from *Cyl. fusiformis*; CffcpA pro.: the promoter region of FCP gene derived from *Cyl. fusiformis*; *Sh ble*: antibiotic Zeocin™ resistance gene isolated from a mutant strain of *Streptomyces verticillus*; (P-ble) denotes the destination vector produced from pCfcp-*ble* [15].

**Figure 5:**
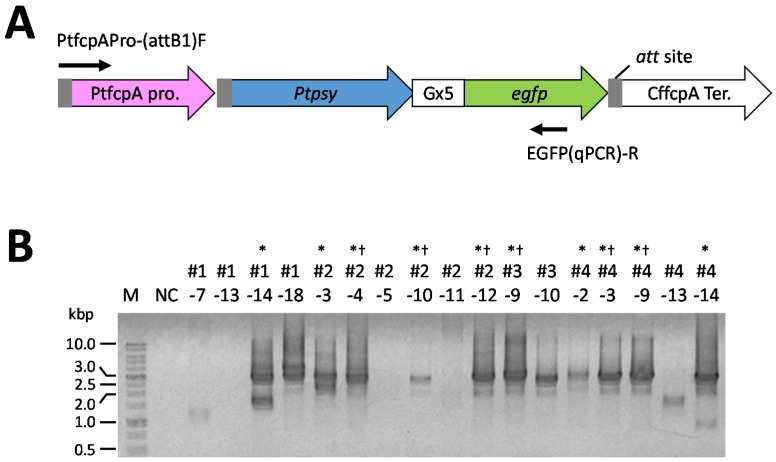
Genomic PCR analysis of the transformed *P. tricornutum*. (**A**) The illustration of transgenes. Arrows indicate the primers used for PCR determination; (**B**) typical electrophoretogram of genomic PCR determination of the *P. tricornutum* transformants. NC shows a negative control without template cells; M: DNA size maker; * indicate that transformants clones possessed the DNA fragment containing the *Ptpsy::Gx5::egfp* with the PtfcpA promoter (approximately 2700 base pairs); † indicates transformants clones used for the determination of total carotenoid content by the spectrophotometric analysis [64].

We constructed the transformation vector shown in Figure 4. In this vector, the PSY gene of *P. tricornutum* was fused to an eGFP gene via a linker sequence (nucleic acid sequence: ggaggcggaggtgga, termed the Gx5 linker). Sixty colonies were obtained on solid f/2 medium containing 500 μg/mL antibiotics after transformation by a microparticle bombardment. We tried to observe eGFP fluorescence in sixty transformants using the fluorescence microscope for screening the transformants that possess eGFP fluorescence. We obtained seventeen transformants that possessed eGFP fluorescence. Subsequently, for the detection of the introduced *Ptpsy::Gx5::egfp* fused to the PtfcpA promoter (fucoxanthin chlorophyll *a*/*c*-binding protein (FCP) gene A derived from *P. tricornutum*), genomic PCR analysis was performed using the colony-forming cells as templates. Six transformants, which showed a single amplified product containing *Ptpsy::Gx5::egfp* with the PtfcpA promoter by genomic PCR analysis were selected (Figure 5). The six transformants were analyzed for the determination of total carotenoid content following the spectrophotometric analysis reported by Ryckebosch *et al.* [64].

The analysis of total carotenoid content in transformants revealed that the total carotenoid content in transformant #2–12 was significantly higher than that in the wild-type cells, while that in transformant #3–9 was significantly lower than that in wild-type cells (Figure S3). No significant difference was found in the carotenoid content between other transformants and the wild-type. Three different types of transformants (#2–4, #2–12 and #3–9) were further analyzed by quantitative reverse transcription-PCR (qRT-PCR) and high performance liquid chromatography (HPLC) analysis.

### 2.4. Analysis of mRNA Expression of the Introduced PSY Gene in the P. tricornutum Transformants

**Figure 6:**
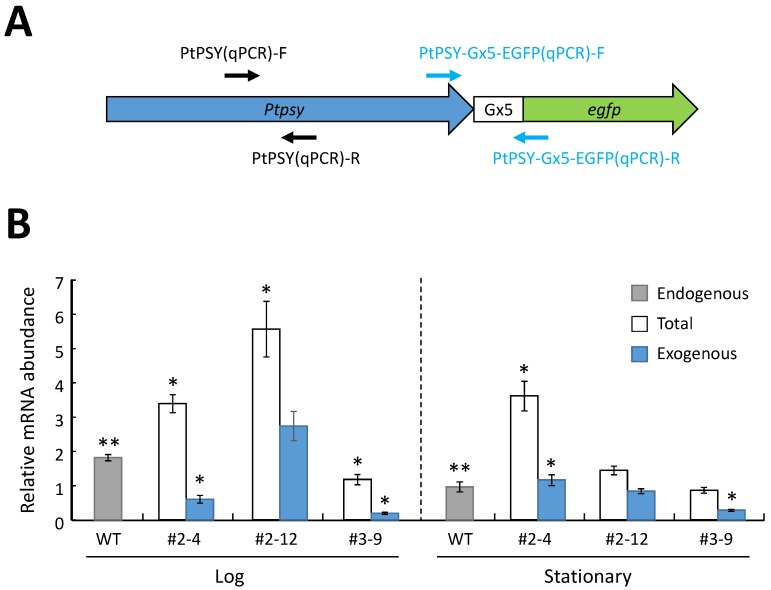
The relative abundances of the PSY mRNA in the wild-type (WT) cells and the transformants cells of *P. tricornutum* at the log and stationary phases determined by qRT-PCR. (**A**) The illustration of the introduced gene. Arrows indicate the primers used for PCR determination; (**B**) PCR determination of PSY mRNA expression. “Exogenous” indicates the abundance of the introduced PSY mRNA. “Total” indicates the total abundance of the intracellular PSY mRNA in transformants cells; “endogenous” indicates the abundance of the intracellular PSY mRNA in the wild-type cells; the data represent the average value of the three independent experiments with standard error; * above bars indicates a significant difference from the endogenous PSY mRNA abundance of the wild-type; ** above bars of the wild-type indicates a significant difference between the endogenous PSY mRNA abundance of the wild-type cells at the log phase and that at the stationary phase.

To confirm the effect of transformation on *P. tricornutum* transformants growth, the growth profiles of the wild-type and transformants were investigated, because total carotenoid content could be remarkably influenced by the growth stage of *P. tricornutum* as described in Rebolloso-Fuentes *et al.* [57]. A marked difference in the transition from the log to stationary phase was not observed (Figure S4). Then, the mRNA expression levels of the PSY gene (*psy*) were investigated in transformants during both the log and stationary phases. The total abundances of the intracellular PSY mRNA (endogenous plus introduced (exogenous)) and of exogenous PSY mRNA were analyzed by qRT-PCR (Figure 6). The abundance of the PSY transcripts in the wild-type cells at the log phase was significantly larger than that at the stationary growth phase (Figure 6B). The exogenous PtfcpA promoter-driven *psy* in the three transformants (#2–4, #2–12 and #3–9) was constitutively expressed during both growth stages (Figure 6B).

In the log phase, the abundances of the exogenous PSY transcripts in the transformants cells (#2–4, #2–12 and #3–9) were approximately 33.2%, 151% and 10.9% of the levels of endogenous PSY transcripts in the wild-type cells, respectively (Figure 6B). The total abundance of the PSY transcripts in transformants #2–4 and #2–12 was significantly larger than that in the wild-type cells, while the abundance in transformant #3–9 was significantly smaller than that in wild-type cells (Figure 6B). In the stationary phase, the abundance of exogenous PSY transcripts in transformants #2–4, #2–12 and #3–9 was approximately 120%, 86.9% and 29.5% the levels in the wild-type cells, respectively (Figure 6B). The total abundance of the PSY transcripts in the transformant cells (#2–4) was significantly larger than that in the wild-type cells (Figure 6B). No significant differences were found in the total abundance of PSY transcripts between the transformants (#2–12 and #3–9) and the wild-type at the stationary phase (Figure 6B).

### 2.5. HPLC Analysis of Extracted Carotenoids

**Figure 7:**
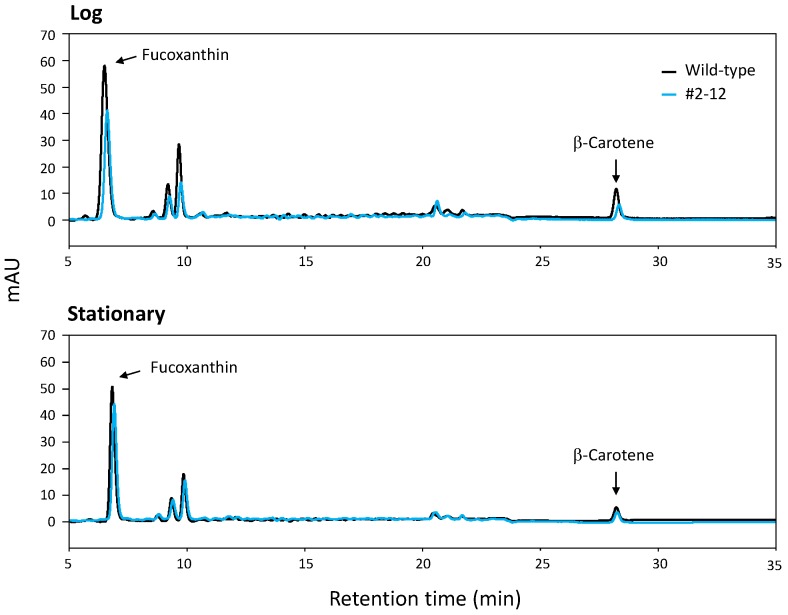
Chromatographic profiles of carotenoids in the wild-type cells and transformant (#2–12) cells of *P. tricornutum* at the log and stationary phases.

Typical HPLC chromatograms that compared the pigment composition of the wild-type and transformant cells are shown in Figure 7. Fucoxanthin and β-carotene peaks were present at approximately 6.5 min and 28.5 min, respectively, in all extracts of the wild-type and the three transformants.

**Figure 8:**
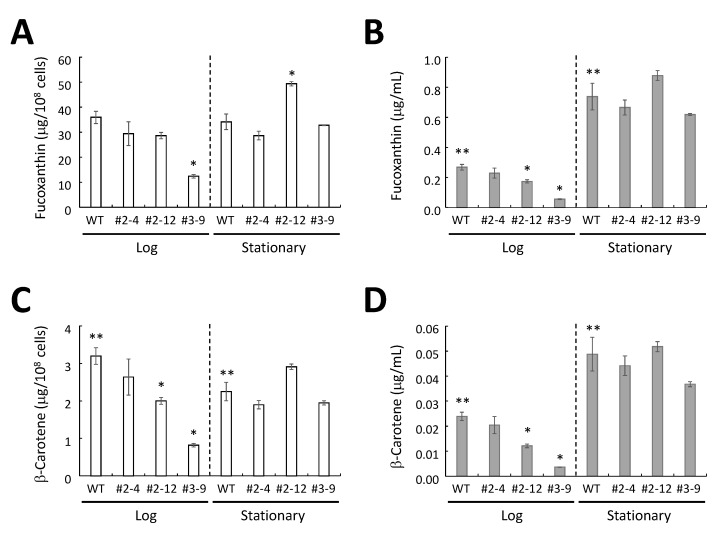
Carotenoid content in *P. tricornutum* in the log and stationary phases. The content of fucoxanthin per cells (**A**) and per mL culture medium (**B**). The content of β-carotene per cells (**C**) and per mL culture medium (**D**). The data represent the average values of the three independent experiments with standard error. * Above bars denotes significantly different from the carotenoid content of the wild-type (WT) cells; ** above bars of the wild-type denotes significantly different between the carotenoid content of the wild-type cells at the log phase and the stationary phase.

There was no significant difference in the amount of fucoxanthin per cell for wild-type cells at the log or stationary phase, whereas the amount of β-carotene per cell of the wild-type at the log phase was significantly larger than that at the stationary phase (Figure 8A,C).

The amounts of fucoxanthin per cell in transformants #2–4 and #2–12 at the log phase were not significantly different from the wild-type levels, whereas the amount of fucoxanthin per cell in transformant #3–9 was significantly lower than the wild-type levels at the log phase (Figure 8A). In the stationary phase, the amounts of fucoxanthin per cell in transformants #2–4 and #3–9 were not significantly different from the levels in the wild-type, whereas the amount of fucoxanthin per cell for transformant #2–12 was approximately 1.45-fold greater than that of the wild-type (Figure 8A). The amounts of β-carotene per cell for transformants #2–12 and #3–9 at the log phase were significantly lower than that of wild-type at the log phase, whereas transformant #2–4 had β-carotene levels at the log phase that were not significantly different from wild-type levels at the log phase (Figure 8C). In the stationary phase, the amounts of β-carotene per cell for the three transformants were not significantly different from the wild-type amount (Figure 8C).

The amounts of β-carotene and fucoxanthin carotenoids per one mL of culture medium increased with the number of cells for the wild-type and three transformants (Figure 8B,D and Figure S4). In the case of transformant #2–12, the amount of fucoxanthin per one mL of medium was approximately 1.19-fold larger than that of the wild-type (Figure 8B).

### 2.6. Localization of eGFP-Fused PSY and the Number of Integrated Copies in the Transformants 

**Figure 9:**
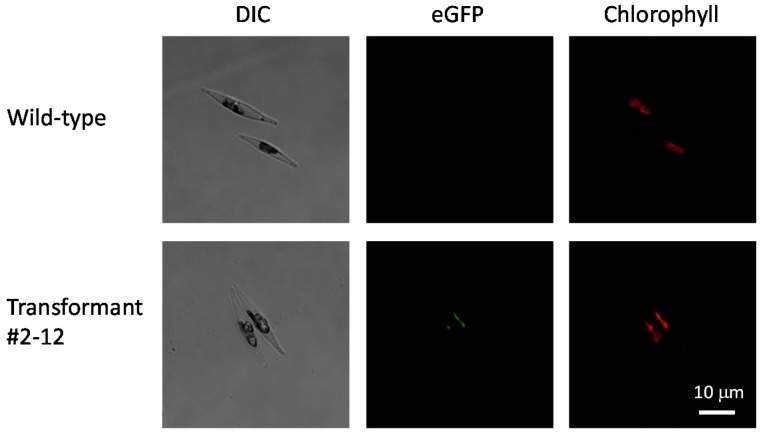
Microscopic image of wild-type and transformed *P. tricornutum*. Images of cells resting in the stationary phase are shown. Differential interference contrast (DIC) images, eGFP fluorescence images and endogenous chlorophyll fluorescence images are presented.

We tried to observe eGFP fluorescence in the plastids in transformant #2–12 using a laser scanning confocal microscope. Transformant #2–12 possessed eGFP fluorescence in its plastids (Figure 9). In addition to localization of PSY, we tried to confirm the number of integrated copies of the introduced *Ptpsy::Gx5::egfp* in the genome of transformant #2–12 by genomic quantitative real-time PCR. The relative fluorescence signal of introduced *Ptpsy::Gx5::egfp* of the transformant was less than half of the fluorescence signal of total *psy* (endogenous plus introduced), suggesting that the transformant might possess one copy of the integrated *Ptpsy::Gx5::egfp* in their genome, whereas the wild-type did not show any fluorescence signal.

## 3. Discussion

We focused on PSY in this study because this enzyme is thought to be a key player in the carotenoid biosynthetic pathway and is found in all photosynthetic organisms and in some non-photosynthetic organisms. In the case of *P. tricornutum* strain NRIA-0065, the mRNA expression level of the PSY gene was relatively low compared to those of the genes for putative regulatory enzymes in the carotenoid biosynthetic pathway, which suggests that PSY may have a key function in the carotenoid biosynthetic pathway of *P. tricornutum*.

The deduced amino acid of the isolated cDNA fragment from the *P. tricornutum* strain NRIA-0065 possessed the typical PSY domains [60]. In plants, PSY enzymes locate in the chloroplasts via the presence of a predicted chloroplast transient peptide at the *N*-terminal region [62]. The plastid targeting conserved motif found in *P. tricornutum* genes that encode nucleus-encoded plastid proteins [63] was missing from the deduced amino acid of the isolated PSY cDNA. *In silico* analysis with the SignalP 4.1 program [65] failed to detect the signal sequence in the deduced amino acid of the isolated cDNA. We have investigated the *N*-terminal regions of potential nucleus-encoded plastid proteins that are involved in the MEP pathway and the carotenoid biosynthetic pathway (twenty-eight proteins, available in Figure 1 and Figure 2). The full length of the plastid targeting the conserved cleavage motif of *P. tricornutum* reported by Gruber *et al.* [63] was found in eight proteins tested in this study (Table S2). In some proteins, the signal peptide cleavage sequence was detected by the SignalP 4.1 program [65] (Table S2). However, we could not find any consensus amino acid motif among the other proteins, including PSY. However, the eGFP-fused PSY in transformants was detected in the plastids, suggesting that PSY of *P. tricornutum* may possesses a novel plastid-targeting sequence.

The HPLC chromatograms of Figure 7 show the two peaks derived from fucoxanthin and β-carotene, which were determined in the wild-type and transformant (#2–12) of *P. tricornutum* NRIA-0065. In addition, three peaks were detected at retention times of approximately 9.5 min, which may be derived from neoxanthin, violaxanthin and diadinoxanthin, based on the HPLC chromatogram result of other *P. tricornutum* strains [40]. The summed area value of the three peaks per cell of transformant #2–12 at the stationary phase was significantly larger than that of the wild-type (Figure S5), suggesting that the amount of carotenoids produced in the late steps of the carotenoid biosynthetic pathway is enhanced by introduction of the PSY gene in transformant #2–12.

The amount and composition of carotenoids seems to be influenced by the growth stage of *P. tricornutum*. The total amount of carotenoids in *P. tricornutum* strain UTEX 640 cells at the stationary phase was reported to be lower than that at the log phase [57]. In the case of *P. tricornutum* strain UTEX 646, the ratio of fucoxanthin to total carotenoids decreased during culture, whereas the ratio of diadinoxanthin to total carotenoids increased [56]. In other microalgae, the cellular carotenoid content, such as lutein in *Chlorella sorokiniana*, increased with cell propagation; however, the cellular lutein content in *Chlorella sorokiniana* decreased at the stationary phase [66]. The variation in β-carotene content was also found in wild-type cells of the *P. tricornutum* strain NRIA-0065, with the amount of β-carotene per cell in the stationary phase significantly lower than the level at the log phase. These results suggest that the growth phase is an important factor for assessing the effects of the introduction of the *psy* gene on carotenoid content in transformants.

In plants and the unicellular alga *Chlamydomonas reinhardtii*, PSY-overexpressing transformants showed an increased accumulation of carotenoids [48,49,50,51,52,53]. It has been reported that the overexpression of PSY in the *P. tricornutum* strain UTEX 646 resulted in a partially correlated increase in the amounts of PSY mRNA and carotenoids [54]. In this study, we also found an increase in the production of fucoxanthin in the PSY-overexpressing transformant #2–12. However, the increased fucoxanthin was not correlated with the amount of PSY mRNA in transformant #2–12 in the same growth phase. Transformant #2–12 showed a significant increase in the amount of fucoxanthin compared to the level in the wild-type at the stationary phase, whereas there was no significant difference in the amounts of PSY mRNA in transformant #2–12 and in the wild-type during the stationary phase. However, in the log phase, the amount of PSY transcript in transformant #2–12 was approximately 3.1-fold higher than the levels observed in the wild-type. We investigated the correlation between the amount of PSY transcript and fucoxanthin content per cell in the wild-type and three transformants at different growth phases (Figure S6). The amounts of PSY transcript during the log phase and fucoxanthin content during the stationary phase were more strongly correlated with each other (*R* = 0.6276, *p* < 0.05) compared to the correlations observed between any other combinations (such as between *psy* at the log phase and fucoxanthin at the log phase, *R* = 0.3345, and between *psy* at the stationary and fucoxanthin at the stationary phase, *R* = 0.3586) (Figure S6). Although regulation of the carotenoid synthetic pathway in diatoms is not fully understood, our results suggest that the step following PSY catalysis might be the rate-limiting step at the log phase, followed by the accumulation of fucoxanthin precursors (e.g., phytoene) in the cell. At the stationary phase, the production of fucoxanthin is enhanced by the use of precursors produced by activation of the rate-limiting step at the log phase. In the case of the red yeast, *Xanthophyllomyces dendrorhous*, which produces astaxanthin through the mevalonate pathway, overexpression of the two genes involved in the early and the late steps of astaxanthin synthesis resulted in a greater enhancement in astaxanthin production than overexpression of the single gene involved in the early step of astaxanthin synthesis [67]. These findings suggest that further enhancement of fucoxanthin production in *P. tricornutum* is possible through genetic engineering of the rate-limiting step at the log phase.

## 4. Experimental Section 

### 4.1. Algal Culture

The Pennales *P. tricornutum* Bohlin (National Research Institute of Aquaculture, Fisheries Research Agency, Mie, Japan, Strain NRIA-0065) was used in this study. Cells were grown at 20 °C under a light cycle of 12 h light and 12 h dark with approximately 90 μmol photons/m^2^/s in liquid f/2 medium [68].

### 4.2. Genomic DNA and RNA-Seq Analysis

Genomic DNA and total RNA were isolated from algae harvested 15 days after inoculation into new liquid f/2 medium at a salinity of 30 practical salinity unit using a DNeasy Plant Mini Kit (QIAGEN Inc., Valencia, CA, USA) and TriPure Isolation Reagent (Roche Diagnostics GmbH, Mannheim, Germany), respectively. To remove DNA from isolated RNA, we used the RNeasy Plant Mini Kit (QIAGEN Inc., Valencia, CA, USA) coupled with a RNase-Free DNase Set (QIAGEN Inc., Valencia, CA, USA). The quality of isolated RNA was checked by the RNA integrity number measured with the Agilent 2100 Bioanalyzer (Agilent Technologies, Inc., Santa Clara, CA, USA). Following isolation of genomic DNA and total RNA, sequence and data analysis were performed using the analysis package provided by Genaris, Inc. (http://www.genaris.co.jp/). Briefly, sequencing of genomic DNA was carried out according to the instructions of the 454 FLX Titanium system (454 Life Sciences, Branford, CT, USA). Sequencing reads were assembled by using Newbler Version 2.8 (454 Life Sciences, Branford, CT, USA). The synthesis of cDNA and sequencing of paired-end reads (2 × 75 bp) were carried out according to instructions accompanying an Illumina HiSeq 2000 sequencer. After trimming the read sequences using a unique algorithm of Genaris, Inc., mapping was performed using TopHat [69], and expression levels were estimated using Cufflinks [70].

### 4.3. Screening of PSY Gene in P. tricornutum Strain NRIA-0065

We searched the PSY gene in the transcriptome database of *P. tricornutum* strain NRIA-0065 using the PSY gene sequence from *P. tricornutum* strain CCAP1055/1, which is available at the Department of Energy Joint Genome Institute web site [9,39,71]. The amino acid sequence of PSY from the NRIA-0065 strain was analyzed by an NCBI-provided Conserved Domain Search [72]. Then, the alignment of amino acid sequences of the PSY genes in various organisms was performed with the program ClustalW using a BioEdit Sequence Alignment Editor [73].

### 4.4. Isolation of PSY Gene

Total RNA was isolated from *P. tricornutum* using an RNeasy Plant Mini Kit (QIAGEN Inc., Valencia, CA, USA) coupled with an RNase-Free DNase Set (QIAGEN Inc., Valencia, CA, USA). Then, cDNA was synthesized with a PrimeScript™ RT Reagent Kit (Perfect Real Time) (Takara Bio Inc., Otsu, Japan). From this synthesized cDNA, PSY cDNA was amplified by PCR with the following primers: forward, PtPSY-(attB4r)F; and reverse: PtPSY-(Gx5)R; shown in Table 1. The forward primer contained the *att* sequence of Gateway^®^ systems (Life Technologies Corporation, Carlsbad, CA, USA) at its 5′ terminal for the construction of the transformation vector. The reverse primer contained a Gx5 linker sequence (ggaggcggaggtgga) at its 3′ terminus to allow joining to the EGFP gene (Figure 4). The amplification of DNA fragments was performed using PrimeSTAR^®^ HS DNA Polymerase (Takara Bio Inc., Otsu, Japan) according to the instructions. The amplicons were purified by agarose gel electrophoresis and a QIAquick^®^ Gel Extraction Kit (QIAGEN Inc., Valencia, CA, USA).

### 4.5. Joint PCR for the Fusion of the PSY Gene and the EGFP Gene

For detection of the introduced PSY gene, an EGFP gene was fused as a tag at the 3′ terminus. The EGFP gene was amplified from the pNICgfp plasmid [15] with the primers: forward, EGFP-(Gx5)F; and reverse, EGFP-(B3r)R; shown in Table 1. The forward primer contained the Gx5 linker sequence at its 5′ terminus to enable fusing to the PSY gene. The reverse primer contained an *att* sequence from Gateway^®^ systems (Life Technologies Corporation, Carlsbad, CA, USA) at its 3′ terminus for the construction of the transformation vector. The amplicons were purified by agarose gel electrophoresis and a QIAquick^®^ Gel Extraction Kit (QIAGEN Inc., Valencia, CA, USA). Excessive amounts of purified amplicons (PSY gene and EGFP gene) were mixed in the PCR reaction mixture, and then, the two fragments were joined by PCR with the primers (forward: PtPSY-(attB4r)F; reverse: EGFP-(B3r)R), which annealed to the termini of the joint-PCR product. Joint PCR amplifications were performed by using PrimeSTAR^®^ GXL DNA Polymerase (Takara Bio Inc., Otsu, Japan) according to the provided instructions. This joint-PCR product was termed *Ptpsy::Gx5::egfp* and was used for the construction of the Gateway^®^ entry vectors (Life Technologies Corporation, Carlsbad, CA, USA).

### 4.6. Transformation Vector Construction

We prepared the transformation vector shown in Figure 4 using a Gateway^®^ cloning system according to the instructions of MultiSite Gateway^®^ Pro (Life Technologies Corporation, Carlsbad, CA, USA). For the construction of the Gateway^®^ entry vectors, DNA fragments containing a promoter region and a terminator region were amplified by PCR using sets of forward and reverse primers (shown in Table 1). Amplicons were then were cloned into plasmids using the Gateway^®^ cloning system (Life Technologies Corporation, Carlsbad, CA, USA) according to the provided instructions. The PtfcpA promoter was amplified from the genomic DNA of the *P. tricornutum* UTEX 646 strain (The Culture Collection of Algae (UTEX), The University of Texas at Austin) with a set of primers (forward: PtfcpA Pro-(attB1)F; reverse: PtfcpA Pro-(attB4)R). The terminator region of the FCP gene was derived from *Cyl. fusiformis* and was amplified from pNICgfp [14] with another set of primers (forward: CffcpTer/F/attB3; reverse: CffcpTer/R/attB2). All DNA fragments were amplified with PrimeSTAR^®^ GXL DNA Polymerase according to the provided instructions. To prepare the Gateway^®^ destination vector, pCfcp-*ble* [15], which contained the antibiotic gene *Sh ble* that was controlled by a *Cyl. fusiformis* FCP gene promoter, was modified by inserting the Gateway^®^ cassette (Life Technologies Corporation, Carlsbad, CA, USA) into a *Kpn*I site of pCfcp-*ble.* This site presents the upstream of the CffcpA pro. region. Finally, we constructed the transformation vector according to the instructions, which was described as pPtfcpA Pro/PtPSY::Gx5::EGFP/Cffcp Ter (P-ble) and is shown in Figure 4.

**Table 1:** Primers designed for the isolation of the PSY gene of *P. tricornutum* (Primer Numbers 1 and 2), for the construction of the transformation vector (3–8), for the genomic PCR analysis (3 and 9) and for the qRT-PCR analysis (10–15).

Primer Number	Name	Sequence (5′–3′)	Annotation
1	PtPSY-(attB4r)F	ggggacaacttttctatacaaagttgctATGAAAGTTTCGACAAAGCTCTG	add *att* to 5′ terminal of *Ptpsy*
2	PtPSY-(Gx5)R	tccacctccgcctccTACTTGATCCAATTGGACC	add Gx5 linker to 3′ terminal of *Ptpsy*
3	PtfcpA Pro-(attB1)F	ggggacaagtttgtacaaaaaagcaggcttaGGGCTGCAGGACGCAATGGAG	add *att* to 5′ terminal of PtfcpA promoter
4	PtfcpA Pro-(attB4)R	ggggacaactttgtatagaaaagttgggtgTCTCGAAACGGCAGACAA	add *att* to 3′ terminal of PtfcpA promoter
5	CffcpTer/F/attB3	ggggacaactttgtataataaagttgTGCGGCCGCATTGCTTGTTG	add *att* to 5′ terminal of CffcpA terminator
6	CffcpTer/R/attB2	ggggaccactttgtacaagaaagctgggtGAGCTCTGGAAGCAT	add *att* to 3′ terminal of CffcpA terminator
7	EGFP-(Gx5)F	ggaggcggaggtggaATGGTGAGCAAGGGCGAGGAGCT	add Gx5 linker to 5′ terminal of *egfp*
8	EGFP-(B3r)R	ggggacaactttattatacaaagttgtTTACTTGTACAGCTCGTCC	add *att* to 3′ terminal of *egfp*
9	EGFP(qPCR)-R	CACGAACTCCAGCAGGACCA	used for genomic PCR analysis
10	PtPSY(qPCR)-F	ATGTTTGGTGTCGACGAACG	detect endogenous and introduced *psy*
11	PtPSY(qPCR)-R	TTGCCACAAACGCTCCAATC	detect endogenous and introduced *psy*
12	PtPSY-Gx5-EGFP(qPCR)-F	GGACGCAAGACATTTCTCAATGG	detect introduced *psy*
13	PtPSY-Gx5-EGFP(qPCR)-R	TCGCCCTTGCTCACCATTC	detect introduced *psy*
14	Q-rps-fw	CGAAGTCAACCAGGAAACCAA	detect *rps*
15	Q-rps-rv	GTGCAAGAGACCGGACATACC	detect *rps*

Additional *att* and Gx5 linker sequences for the construction of the transformation vector are shown in lowercase.

### 4.7. Microparticle Bombardment and Selection of Transformants

The plasmids were introduced into *P. tricornutum* cells using the Biolistic PDS-1000/He particle delivery system (Bio-Rad Laboratories, Hercules, CA, USA) according to a method that has been previously reported [10,12,20]. Approximately 1 × 10^8^ cells were spread, centrally covering one third of a plate of solid f/2 medium containing 0.5% agarose HGS (Nacalai Tesque, Kyoto, Japan) approximately 1 h prior to bombardment. The plate was positioned at the second level (6 cm from the stopping screen) within the Biolistic chamber for bombardment. Three milligrams of M17 tungsten particles (1.1 μm diameter, Bio-Rad Laboratories, Hercules, CA, USA) were coated with 5 μg of the transformation vector in the presence of CaCl_2_ and spermidine for five shots, as described by the manufacturer. Cells were then bombarded with 600 ng of DNA-coated tungsten particles using two-ply, 650-psi rupture disks. After bombardment, the cells were incubated in liquid f/2 medium for 24 h under constant illumination. The cell suspensions of *P. tricornutum* were spread onto plates of solid f/2 medium containing 0.5% agarose HGS supplemented with 500 µg/mL Zeocin™ (InvivoGen, San Diego, CA, USA). After 5 weeks of the selective incubation under standard growth conditions, individual colonies were picked with a platinum loop and suspended into liquid media with antibiotic.

### 4.8. Microscopic Analysis

To observe the fluorescence from eGFP in the transformed cells, wild-type and transformants resting in stationary phase were analyzed on an Olympus IX-81 and FV-1000 laser scanning confocal microscope with Olympus FV-10 software (Olympus Corporation, Tokyo, Japan). The eGFP fluorescence was analyzed using a 473-nm laser and a 490/525-nm band pass filter. Endogenous chlorophyll fluorescence was analyzed using a 543-nm laser and a 560/620-nm band pass filter. Images were analyzed using the OLYMPUS FLUOVIEW Ver. 4.2 software (Olympus Corporation, Tokyo, Japan).

### 4.9. PCR Analysis of the Introduced Gene and Promoter in Transformed Cells

To determine whether the *Ptpsy::Gx5::egfp* gene with the PtfcpA promoter was successfully introduced into the colony-forming cells, the regions containing the promoter and the *Ptpsy::Gx5::egfp* gene were amplified by using the cells as a template. A primer set of PtfcpA Pro-(attB1)F forward primer and EGFP(qPCR)-R reverse primer was used for the genomic PCR analyses, along with Tks Gflex^®^ DNA Polymerase (Takara Bio Inc., Otsu, Japan), according to the provided instructions. Then, amplicons were analyzed by agarose gel electrophoresis.

### 4.10. Preparation of Cells Used for qRT-PCR and Carotenoid Analyses of Transformants

*P. tricornutum* wild-type and transformants cells at the stationary growth phase were inoculated into fresh liquid f/2 medium at final concentrations of 2 × 10^4^ cells/mL and were propagated at 20 °C under a light cycle of 12 h light and 12 h dark with approximately 90 µmol photons/m^2^/s. The cell density in each culture was determined by sampling the cell suspension from the well-stirred culture. The cells were counted under a microscope using a hemocytometer. This count was repeated 3 times, and the mean cell density in the culture was calculated. Approximately 2 × 10^7^ cells at the log phase and the stationary phase were sampled from the cultures for the analyses of qRT-PCR and carotenoid analyses.

### 4.11. Quantitative RT-PCR

Total RNA was isolated from approximately 2 × 10^7^ transformed cells using an RNeasy Plant Mini Kit (QIAGEN Inc., Valencia, CA, USA). Reverse transcription (RT) was performed using a PrimeScript^®^ RT Reagent Kit (Perfect Real Time) (Takara Bio, Otsu, Japan). The qRT-PCR experiments were performed in triplicate in a Thermal Cycler Dice^®^ Real Time System Single MRQ (Takara Bio Inc., Otsu, Japan) using 2 µL of the cDNA mixture added as a template and SYBR^®^ Premix Ex Taq™ II (Tli RNaseH Plus) (Takara Bio Inc., Otsu, Japan). Cycling conditions were as follows: 30 s at 95 °C for denaturing and 40 cycles for melting (5 s at 95 °C) plus annealing coupled with extension (30 s at 60 °C). Each qRT-PCR measurement was carried out in triplicate using primers for the quantification of intracellular PSY mRNA (forward: PtPSY(qPCR)-F; reverse: PtPSY(qPCR)-R) and that of the introduced PSY mRNA (forward: PtPSY-Gx5-EGFP(qPCR)-F; reverse: PtPSY-Gx5-EGFP(qPCR)-R), which are summarized in Table 1. The ribosomal protein small subunit 30S gene (*rps*) has been shown to be constitutively expressed under various growth conditions [74] and was used for standardization (copy number of PSY mRNA divided by the copy number of RPS mRNA) of the different samples.

### 4.12. Carotenoid Extraction and Analysis

Freeze-dried cells (approximately 2 × 10^7^ cells) of *P. tricornutum* at both the log and stationary growth phases were extracted with 100% acetone at room temperature and centrifuged, and the supernatant was collected a total of 6 times (for a total volume of acetone of 650 μL). Following acetone extraction, the residual pellet was extracted with 100% methanol at room temperature and centrifuged, and then, the supernatant was collected 4 times (the total volume of methanol was 400 μL). The supernatant was evaporated under N_2_ gas, and the pellet was dissolved in 600 μL methanol. Then, the re-dissolved samples were centrifuged and the supernatants were analyzed. HPLC analysis was performed on a Varian 320-MS LC/MS equipped with a diode array detector (Agilent Technologies, Inc., Santa Clara, CA, USA) using a CrestPack C18T-5 column (4.6 mm× 250 mm, JASCO Corporation, Tokyo, Japan) with heating at 40 °C, a flow rate of 1 mL/min and a final injection volume of 10 μL. The two mobile phases used included Solvent A, acetonitrile: methanol: H_2_O (15:3:2 (v/v)) containing 0.1% (w/v) ammonium acetate, and Solvent B, ethyl acetate: methanol (3:7 (v/v)) containing 0.1% (w/v) ammonium acetate. The gradient applied was: 0–20 min 100%–0% A and 0%–100% B; 20–35 min 100% B. Standards of β-carotene and fucoxanthin were supplied by Wako Pure Chemical Industries, Ltd. (Osaka, Japan). Pentaplicate measurements were taken, and the mean was then calculated.

### 4.13. Genomic Quantitative Real-Time PCR

Genomic DNA was isolated from approximately 1 × 10^8^ transformed cells or wild-type cells using a DNeasy Plant Maxi Kit (QIAGEN Inc., Valencia, CA, USA). The genomic quantitative real-time PCR experiments were performed in triplicate in a Thermal Cycler Dice^®^ Real Time System Single MRQ (Takara Bio Inc., Otsu, Japan) using 2 ng of the genomic DNA mixture added as a template and SYBR^®^ Premix Ex Taq™ II (Tli RNaseH Plus) (Takara Bio Inc., Otsu, Japan). Cycling conditions were as follows: 30 s at 95 °C for denaturing and 40 cycles for melting (5 s at 95 °C) plus annealing coupled with extension (30 s at 60 °C). Each PCR measurement was carried out in triplicate using primers for the quantification of total *psy* (forward: PtPSY(qPCR)-F; reverse: PtPSY(qPCR)-R) and that of the introduced *Ptpsy::Gx5::egfp* (forward: PtPSY-Gx5-EGFP(qPCR)-F; reverse: PtPSY-Gx5-EGFP(qPCR)-R), which are summarized in Table 1.

### 4.14. Statistical Analyses

All data were obtained from three independent experiments. Experimental results were expressed as the mean values ± standard errors. Statistical analyses were performed using Student’s *t*-test for two group mean values (the wild-type and the transformants), and statistical significance was achieved when *p* < 0.05.

## 5. Conclusions

With the aim of increasing the accumulation of carotenoids, we attempted to engineer *P. tricornutum* by transforming a tagged PSY gene isolated from *P. tricornutum*, which had shown lower expression levels among the genes involved in the carotenoid biosynthetic pathway. In the log phase, the *P. tricornutum* transformants containing the PtfcpA promoter-controlled PSY gene showed a significant increase in the abundance of PSY transcripts compared to the wild-type levels. We found that the amount of PSY mRNA transcript present during the log phase might contribute to the increase in carotenoids in the transformants at the stationary phase.

## Supplementary Files

Supplementary File 1
