# Supplementary material for: Effect of an Introduced Phytoene Synthase Gene Expression on Carotenoid Biosynthesis in the Marine Diatom Phaeodactylum tricornutum
Source: Mar Drugs. 2015 Aug 20;13(8):5334–57. doi: 10.3390/md13085334 (PMC4557025; doi:10.3390/md13085334)
Supplement: Supplementary File 1 [file marinedrugs-13-05334-s001.docx]

Supplementary Information

**Table S1.** Primers designed for the phylogenetic analyses.

| **Name** | **Sequence (5′-3′)** | **Annotation** |
| --- | --- | --- |
| Dino5′UF | CAACCTGGTGATCCTGCCAGT | Amplification and sequence of 18S rDNA. Litaker *et al*. [1] |
| 18ScomR1 | CACCTACGGAAACCTTGTTACGAC | Amplification of 18S rDNA.  Zhang *et al*. [2] |
| G10R | CCGCGGCTGCTGGCACCAGAC | Sequence of 18S rDNA.  Litaker *et al*. [1] |
| ITSA | GTAACAAGGTHTCCGTAGGT | Amplification and sequence of ITS1-5.8S rDNA-ITS2. Sato *et al*. [3] |
| ITSB | AKATGCTTAARTTCAGCRGG | Amplification and sequence of ITS1-5.8S rDNA-ITS2. Sato *et al*. [3] |

**Table S2.** *N*-terminal regions of putative proteins in the MEP pathway and the carotenoid biosynthetic pathway.

| **Protein** | **40 Amino Acid Sequences in the *N*-terminal Regions** | **Motif** *^6^ |
| --- | --- | --- |
| DXS | MRLSSALFLLITPTV**AA**-**FAP**RASLLVRNTNTFVKAEGTNG |  |
| DXR | MRLTELSVSLIVGS**AAA**-**FAP**TTGISATRTTSASTVRLQLV | Yes |
| MCT | MIALRISFLFVLWVLCL**SSA**•**FA**-**F**LTVQRPTVVSRICRESL | Yes |
| CMK *^1^ | MYFASTYVLLLAALSRE**A**V**A**-**FTG**NHFPIFNHQSAEVATTS |  |
| CMK *^2^ | MEKNRRATGSVSRKRVGAFVCFSLRCLCSPIVTAMGLERV |  |
| MDS | MKSSS•FLLYSILSAAIVPYAAFVVGGVTPGGRYLRTITAA | Yes |
| HDS | MKFLGLTAIMLTGLATSPVQ**A**-**FVP**RMASFGRLQTTASMSS |  |
| HDR | MRFASSAVVFMAVASTVT**A**-**FQQ**TAFKPSRISIVVQQSTSA |  |
| IDI | MIIL**GAA**•**WIG**RVAGLAPSAKSRQFMVAQSWGLRAIKDRCA | Yes |
| GGDS | MRVSLLALVSLTTLSS**ASA**-**FVA**PSTFHFRPRVTLQSTAAT | Yes |
| PSY1 | MKVSTKLCYAVCTCAPLLTRAWIGNKMHFSGVPGTNTYGL |  |
| PDS1 | MMFHYKT**GSS**•**WFL**LLSASITTTLT-T**TT**MTTTHAFAPHTRL | Yes |
| PDS2 | MAVLSCWNAWAEAFAPNTNVPFHRIAKAQSSLSIVQAPDF |  |
| ZDS | MSSCRKPNEIEIDGSKGEGGGQILRNSISYATILRKPIRI |  |
| CRTISO1 | MRTTTMANISKDRLTGRFLAFLLLVLANKET**SS**-**FCV**QSGY |  |
| CRTISO2 | MLVESKKSRDGSRTSSRSLDTKTHCVCSTSKQNSVRPANT |  |
| CRTISO3 *^3^ | MFAISSQLTLTLVGHLILLHMMENSAIC**SA**-**FVP**ASQRTTF |  |
| CRTISO4 | MRFSERSLIACAICSI**SSA**-**FVP**IIHTPQHQSPRTTRHQFT | Yes |
| CRTISO5 | MRRHCLLSALLLLSGPGLDQ**S**-R**SF**RVVAFQTSSAPRSSLS |  |
| CRTISO-like | MQQITSMISPEAFLPLATTLPKIPFWILAPVLIWVGFLVW |  |
| LCYB | MKVILADQNVDKVWPANYGVWQDEWQSVVDRFQAAGVPFV |  |
| LUT-like1 | MYSITFLSLTAVVVVACFFPKRTQ**S**-**LVV**PFPSSAVRCCFG |  |
| LUT-like2 | MRSSDYSRTPRWESFVVLAWATASAVLVGNVFQQALPQPV |  |
| ZEP1 | MKFSTTV**SSA**•**LFL**IASVSTTT**S**-**FTP**VQSFGVHRRNLLVTP | Yes |
| ZEP2 | MSVAWFAFDVVRICVHASRPRVTTRYVGYHALIRVSGIPP |  |
| ZEP3 *^4^ | MKRSCSIVTILYVATTVR**A**-**FAP**APLVQSSCFFQRQPTTTA |  |
| VDE | MKFLGVTSLLCLWSVVNR-E**NV**SEAFAPRHQSLSRPSSRTT |  |
| VDL1 *^5^ | MRFAWVVAAGVVLTTTTQ**A**-**LVP**LDCTGMGETRTSGIRPIR |  |
| VDL2 | MKRATRKRTLAATLWIAMSSVT**G**-S**GP**GRTAAFAPSGNNNG |  |

*^1^ The potential translation start site refers to NCBI Reference Number XP_002178363; *^2^ the potential translation start site refers to RNA-seq analysis; *^3^ the sequence is XP_002182606 due to the lack of the
*N*-terminal region in RNA-seq analysis; *^4^ the sequence is XP_002178367 due to the lack of the *N*-terminal region in the RNA-seq analysis; *^5^ the sequence is XP_002180635 due to the lack of the *N*-terminal region in RNA-seq analysis; *^6^ “Yes” means the presence of amino acid sequences in the plastid targeting cleavage site reported by Gruber *et al*. [4]; bold letters show the consensus motif reported by Gruber *et al*. [4]; • represents the cleavage site; grey: signal peptide cleavage sequence predicted by SignalP 4.1 [5]; “-” represents the cleavage site.


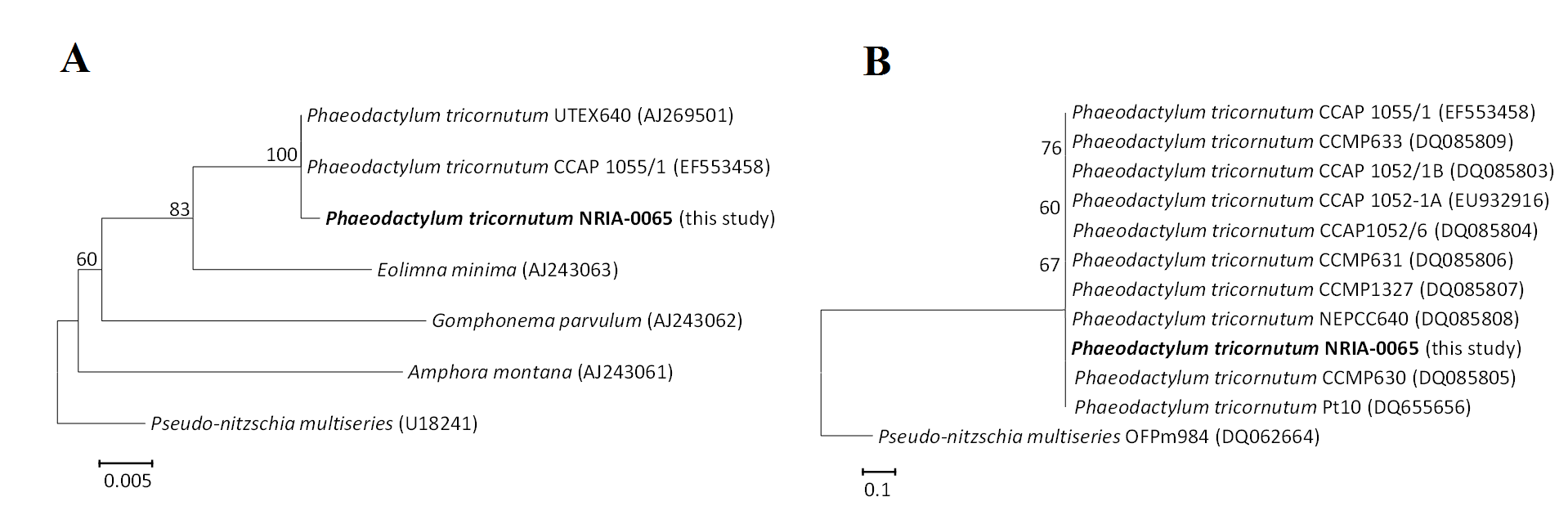


**Figure S1.** Maximum likelihood analyses of the 18S rDNA sequence (**A**) and ITS1-5.8S rDNA-ITS2 sequence (**B**) from *P. tricornutum*
NRIA-0065. The numbers at nodes on the branches represent bootstrap values (1000 replicates). *Pseudo-nitzschia multiseries* was used as an outgroup species for both the 18S rDNA and ITS1-5.8S rDNA-ITS2 datasets. Accession numbers of genes are shown in parentheses. The bar represents nucleotide substitutions per position.

**Figure S2.** Multiple alignment of amino acid sequences of PSY of various organisms. *Arabidopsis thaliana* (NCBI reference sequence: NP_197225.1), *Chlamydomonas reinhardtii* (NCBI reference sequence: XP_001701192.1), *Dunaliella salina* (GenBank: AAT46069.1), *Nostoc* sp. PCC 7120 (NCBI reference sequence: WP_010996001.1), *Oryza sativa* Japonica Group (GenBank: AAK07735.1), *Solanum lycopersicum* (NCBI reference sequence: NP_001234812.1), *Synechocystis* sp. PCC 6803 (NCBI reference sequence: WP_010872477.1), *Thalassiosira pseudonana* (NCBI reference sequence: XP_002290727.1), *Zea mays* (NCBI reference sequence: NP_001108124.2). Highlighted in grey: trans-isoprenyl diphosphate synthase domain; Framed amino acids: aspartate rich regions and substrate-Mg^2+^-binding sites (DXXXD).

**Figure S3.** Total carotenoid content in wild-type (WT) and transformants of *P. tricornutum* at the stationary phase. The carotenoid content in the wild-type and transformants was measured by spectrophotometric analysis [6]. The data represent the average values of the three replicates with standard deviation; * above bars denotes significantly different from the carotenoid content of the wild-type cells.

**Figure S4.** Growth of wild-type and three transformants. The data represent the average value of the three measurements in three independent experiments. WT: wild-type. #2–4, #2–12 and #3–9 are transformants; arrows show the collecting point of cells for qRT-PCR analysis and HPLC analysis.

**Figure S5.** Putative contents of neoxanthin, violaxanthin and diadinoxanthin in *P. tricornutum* at the log and stationary phases. The summed area value of the three peaks was detected at a retention time of approximately 9.5 min, which may be derived from neoxanthin, violaxanthin and diadinoxanthin per cell (**A**) and per one mL of culture medium (**B**). WT: wild-type; #2–4, #2–12 and #3–9 are transformants; * above bars denotes significantly different from the area values of the wild-type cells; ** above bars of the wild-type indicates a significant difference between the area values of the wild-type cells at the log phase and at the stationary phase.

**Figure S6.** The correlation between the amount of PSY transcript and fucoxanthin content per cell at different growth phases. Circle, square, triangle and diamond shows the data of wild-type, transformants #2–4, #2–12 and #3–9 in three independent cultures, respectively. * *p* < 0.05.

Supplementary Methods

Phylogenetic Analyses of P. tricornutum Strain NRIA-0065

To clarify the phylogenetic position of *P. tricornutum* strain NRIA-0065, we carried out phylogenetic analyses of this strain. Cells were collected by centrifugation (10 min, 3000× *g*); then, cell pellets were washed with ultra-pure water. These cells were used as a template to amplify the nuclear-encoded 18S rDNA and ITS1-5.8S rDNA-ITS2. Amplifications were performed in PCR using a MightyAmp^®^ DNA Polymerase Ver.2 (Takara Bio Inc., Otsu, Japan). Using primer sets of forward: Dino5′UF [1] and reverse: 18ScomR1 [2] for 18S rDNA and forward: ITSA [3] and reverse: ITSB [3] for ITS1-5.8S
rDNA-ITS2, partial regions of 18S rDNA and ITS1-5.8S rDNA-ITS2 were amplified. The residual primers and dNTPs were removed from amplicons using a High Pure PCR Clean up Micro Kit (Roche Applied Science, Mannheim, Germany), and then, the sequencing of amplicons was performed with an ABI PRISM^®^ 3100 genetic analyzer (Life Technologies Corporation, Carlsbad, CA, USA) using a BigDye^®^ Terminator v3.1 Cycle Sequencing Kit (Life Technologies Corporation, CA, USA) according to the manufacturer’s instructions. Both strands of each amplicon were sequenced using the primer set of Dino5′UF forward primer and G10R reverse primer [1] for 18S rDNA and the primer set of the initial amplification for ITS1-5.8S rDNA-ITS2. Phylogenetic analyses were conducted using the obtained sequences of partial 18S rDNA (580 bases) and ITS1-5.8S rDNA-ITS2 (471 bases) using a maximum likelihood (ML). We used MEGA 6 software [7] to determine the most appropriate model for molecular evolution using a corrected Akaike information criterion. The ML analyses were constructed with MEGA 6 software [7] with model parameters (18S rDNA: Tamura-Nei model with gamma distribution, ITS1-5.8S rDNA-ITS2: Kimura two-parameter model) fixed according to values determined with 1000 replications of the bootstrap algorithm [8].

Measurement of Total Carotenoid Content

Total carotenoid content in wild-type and transformants was measured according to
Ryckebosch *et al*. [6]. In brief, cells at the stationary phase were harvested. The extraction of carotenoids was described in the “Experimental Section”. Absorbance was measured at 470, 665 and 652 nm using a spectrophotometer DU730 (Beckman Coulter, Inc., Bream, CA, USA). The following equation was used to calculate the amount of carotenoids:

| $C_{a}=\left( 15.65A_{665} \right)-(7.34A_{652})$ |
| --- |
| $X_{carotenoids}=\frac{\left( 1000C_{470} \right)-\left( 2.86C_{a} \right)}{221}\times\mathrm{DF}\times V$ |

where *C*_a_ is the concentration of chlorophyll *a* and *A*_665_, *A*_652_ and *A*_470_ are the absorbance at 665 nm,
652 nm and 470 nm, respectively. *X*_carotenoids_ is the amount of carotenoids extracted (μg); DF is the dilution factor; and *V* is the volume of extracted carotenoids solution.

References

1. Litaker, R.W.; Steidinger, K.A.; Mason, P.L.; Landsberg, J.H.; Shields, J.D.; Reece, K.S.;
Haas, L.W.; Vogelbein, W.K.; Vandersea, M.W.; Kibler, S.R.; *et al*. The reclassification of *Pfiesteria shumwayae* (Dinophyceae): *Pseudopfiesteria*, gen. nov. *J. Phycol.* **2005**, *41*, 643–651.

2. Zhang, H.; Bhattacharya, D.; Lin, S. Phylogeny of dinoflagellates based on mitochondrial cytochrome *b* and nuclear small subunit rDNA sequence comparisons. *J. Phycol.* **2005**, *41*,
411–420.

3. Sato, S.; Nishimura, T.; Uehara, K.; Sakanari, H.; Tawong, W.; Hariganeya, N.; Smith, K.;
Rhodes, L.; Yasumoto, T.; Taira, Y.; *et al*. Phylogeography of *Ostreopsis* along west Pacific coast, with special reference to a novel clade from Japan. *PLoS ONE* **2011**, *6*, e27983, doi:10.1371/journal.pone.0027983.

4. Gruber, A.; Vugrinec, S.; Hempel, F.; Gould, S.B.; Maier, U.G.; Kroth, P.G. Protein targeting into complex diatom plastids: functional characterisation of a specific targeting motif. *Plant Mol. Biol.* **2007**, *64*, 519–530.

5. Petersen, T.N.; Brunak, S.; von Heijne, G.; Nielsen, H. SignalP 4.0: Discriminating signal peptides from transmembrane regions. *Nat. Methods* **2011**, *8*, 785–786.

6. Ryckebosch, E.; Muylaert, K.; Eeckhout, M.; Ruyssen, T.; Foubert, I. Influence of drying and storage on lipid and carotenoid stability of the microalga *Phaeodactylum tricornutum*. *J. Agric. Food Chem.* **2011**, *59*, 11063–11069.

7. Tamura, K.; Stecher, G.; Peterson, D.; Filipski, A.; Kumar, S. MEGA6: Molecular evolutionary genetics analysis version 6.0. *Mol. Biol. Evol.* **2013**, *30*, 2725–2729.

8. Efron, B. *The Jackknife, the Bootstrap and other Resampling Plans*; Society for Industrial and Applied Mathematics: Philadelphia, PA, USA, 1982; p. 96.

© 2015 by the authors; licensee MDPI, Basel, Switzerland. This article is an open access article distributed under the terms and conditions of the Creative Commons Attribution license (http://creativecommons.org/licenses/by/4.0/).
